# Beyond the “I” in the Obesity Epidemic: A Review of Social Relational and Network Interventions on Obesity

**DOI:** 10.1155/2013/348249

**Published:** 2013-08-26

**Authors:** Janette S. Leroux, Spencer Moore, Laurette Dubé

**Affiliations:** ^1^School of Kinesiology and Health Studies, Queen's University, 28 Division Street Kingston, ON, Canada K7L 3N6; ^2^Department of Community Health and Epidemiology, Queen's University, Kingston, ON, Canada K7L 2N8; ^3^Desautels Faculty of Management, McGill University, 1001 rue Sherbrooke Ouest, Montreal, QC, Canada H3A 1G5

## Abstract

*Background*. Recent research has shown the importance of networks in the spread of obesity. Yet, the translation of research on social networks and obesity into health promotion practice has been slow. *Objectives*. To review the types of obesity interventions targeting social relational factors. *Methods*. Six databases were searched in January 2013. A Boolean search was employed with the following sets of terms: (1) social dimensions: social capital, cohesion, collective efficacy, support, social networks, or trust; (2) intervention type: intervention, experiment, program, trial, or policy; and (3) obesity in the title or abstract. Titles and abstracts were reviewed. Articles were included if they described an obesity intervention with the social relational component central. Articles were assessed on the social relational factor(s) addressed, social ecological level(s) targeted, the intervention's theoretical approach, and the conceptual placement of the social relational component in the intervention. *Results*. Database searches and final article screening yielded 30 articles. Findings suggested that (1) social support was most often targeted; (2) few interventions were beyond the individual level; (3) most interventions were framed on behaviour change theories; and (4) the social relational component tended to be conceptually ancillary to the intervention. *Conclusions*. Theoretically and practically, social networks remain marginal to current interventions addressing obesity.

## 1. Introduction

 Obesity is recognized as one of the gravest threats to public health of our time [1]. Current intervention strategies meant to curb the spread of obesity have been ineffective [2, 3]. Addressing the magnitude of the obesity epidemic requires the development of multilevel and cross-sectoral interventions [4]. Genetic, biological, and psychological factors interact with obesogenic environmental conditions to promote inactivity, poor nutrition, and, resultantly, widespread weight gain [5–7]. Social epidemiological research has highlighted the importance of social determinants, such as gender, age, socioeconomic status and ethnicity, on health. Interventions on individual behaviors and choices fail, however, to account for the social relational conditions that influence personal choices and behaviors and limit the effectiveness and impact of obesity interventions [8, 9]. There is growing consensus on the need to shift the paradigm for addressing the prevalence of obesity to social domains beyond the individual [8, 10, 11]. The degree to which social relational constructs have been integrated into obesity interventions remains unclear. 

A number of social relational constructs have gained prominence in recent social epidemiological research on obesity. These constructs included social cohesion, collective efficacy, trust, social capital, social support, and social networks. Social cohesion describes the trust, respect, and participation within a community and has been conceptualized as a social-structural, cultural condition that impacts health through community integration [12]. Collective efficacy may refer to the norms and networks of relationships that enable collective action and a culture of informal social control and social cohesion, whereby people are united and willing to act for the good of the community [13]. Collective efficacy has been proposed as a constraint on unhealthy behaviors [13] and a means through which a community is able to operate as a unit to procure social trust, security, and resources within society at large [14]. Depending on the perspective, social capital can be considered as a communitarian- or network-driven phenomenon. A communitarian definition would conceptualize social capital as comprising elements of a sense of belonging, participation and civic engagement, reciprocity and cooperation, and community trust. A network-based definition of social capital would consider the availability and accessibility of resources within an individual's social network. Independent of these differences in definitions and measurements, both approaches have yielded associations with health outcomes [15], including obesity [16]. Social networks can be defined as a web of social relationships and are characterized by overall structure, as well as the individual ties of which it is comprised. More recent sophisticated methods of social network analysis have revealed a social patterning of a number of health outcomes. Christakis and Fowler [17] demonstrated the spread of obesity in social networks using longitudinal data and validated old and new interest in harnessing the potential of social networks in relation to population health. Social support, which is categorized by instrumental and financial, informational, appraisal, and emotional forms of support, is conceptualized as a psychosocial mechanism which connects social relationships and individual health through psychological, behavioral, and physiological pathways [18]. 

Findings on the impact of social relationships on obesity encourage the shift to interventions beyond the “I”, or individual level and toward interpersonal dynamics by which behaviours are shared, norms formed, and resources (e.g., information, support) exchanged. The objective of this review is to examine the current state of social relational interventions on obesity and characterize the degree to which these interventions have addressed key social relational constructs in intervention planning and implementation. 

## 2. Methods

### 2.1. Search Strategy, Search Terms, and Search Criteria

 To identify the types of interventions targeting obesity from a social influence perspective, we conducted a systematic literature review on social relational interventions targeting obesity. PubMed, Web of Knowledge, CINAHL, EMBASE, TRoPHI, and OVID MEDLINE were all searched in January 2013. The searches were restricted to full-text, English-language articles. A Boolean search strategy was employed with the search designed to identify articles with the following sets of terms in their title or abstract: (1) social dimensions: social capital, social cohesion, collective efficacy, social support, social networks, or trust; (2) experimental conditions: intervention, experiment, program, trial, or policy; and (3) obesity.

### 2.2. Inclusion Criteria, Review Methods, and Data Synthesis

 Duplicate articles were removed from the database of articles. From this pool, articles were included in the next stage if they described an obesity-focused intervention among the general population, and the social relational construct was central enough to the intervention that it was included in the title or abstract. Studies that were removed from the original pool of articles included those that addressed eating disorders, chronic diseases, or postpartum women. These criteria were applied independently by two researchers. Disagreements on the inclusion of specific articles were discussed and resolved by consensus. 

 The final selection of studies was reviewed to assess and characterize each study by (1) social relational construct addressed, (2) social ecological level targeted, (3) theoretical approach used to guide the intervention, and (4) the placement of social relational construct on the intervention's conceptual pathway. The social relational constructs were social capital, social cohesion, collective efficacy, social support, social networks, and trust. The social ecological model was used as a framework by which to determine the social ecological level(s) targeted by the intervention [19] and included individual, interpersonal, organizational, community, and political levels. To distinguish between interpersonal-level interventions and individual-level interventions that included an interpersonal component, the ensuing criteria were followed: a study was considered an interpersonal intervention if it involved one or more members of a study participant's existing social network. The theoretical rationale for each intervention was garnered from each study when provided. 

 A conceptual typology was developed based on the role of the social relational construct in the intervention. The typology identified three potential roles for social relational constructs to play in an obesity intervention: intervention target, delivery channel, and ancillary resource. The intervention target was defined as a modifiable social relational construct lying directly on the intervention pathway. The delivery channel was defined as the functional or structural means of delivering the intervention, or a vehicle meant to facilitate the intervention. The ancillary resource was defined as a reinforcing but noncentral dimension of the study. Ancillary resources might contribute to the uptake or success of the intervention but was not a critical component of the delivery channel or intervention target. For example, an ancillary resource would be one where the intervention was seeking to change health behavior and delivers the program in a group setting which facilitates group cohesion and social support among study participants. 

## 3. Results

 Database searches using title criteria yielded 664 titles. Application of the inclusion criteria narrowed results to 30 studies. Interrater reliability of the 79 full-text articles to the 30 final studies was calculated as Cohen's kappa coefficient (Kappa = 0.80, SE: 0.07) [20].  Table 1 provides a comprehensive overview of each study, organized by social relational construct (type, modality, and measurement), intervention type, theoretical explanation or reference, social ecological level the intervention was targeting, and type of social relational construct conceptual pathway placement. 

Table 2 provides a descriptive overview of these 30 studies.

The vast majority of studies (*N* = 22) featured social support as the social relational construct, whether alone (*N* = 20) or in combination with social capital [21] or social cohesion [22]. The ways that social support was incorporated into interventions ranged widely between interventions related to the provisioning of professional advice and telephone consultation, to motivational workbooks, to the inclusion of a family or friend in the program itself, and to instructional sessions or interactive group sessions. The measurement of social support varied considerably across interventions from no measures, formal survey instruments, to informal qualitative assessments. Ten of the twenty studies did not measure social support despite the fact that the construct was included in the abstract or description of the intervention. Two studies featured social cohesion, one of which specifically examined family cohesion [23] and the other social cohesion in a weight-loss group [24]. Social cohesion was measured in both studies with the use of (different) questionnaire scales. Six studies featured social networks as the main social relational construct, although the way in which social networks were incorporated varied considerably. Social networks were observed to be used as a study recruitment strategy [25], a structure for transmitting health programs and social influence [26–29], and a changeable entity which might evolve in the intervention [30]. Three of the six studies measured the social network component, which included a study-specific survey [30], a qualitative report of social influence [26], and a quantitative report of social influence [28]. There were no studies that addressed social trust, collective efficacy, or social capital exclusively. Interventions focused primarily on the individual level but occasionally spanned into the interpersonal realm due to the use of smaller supplementary components. For example, a school-based intervention program tailored for adolescent girls sent home four parent newsletters/progress reports which reported their children's time spent in physical activity, sedentary behaviours, and self-reported fruit and vegetable consumption. In addition, the newsletters included information meant to increase awareness and encourage parents to support their children's physical activity and dietary behaviors [31]. Such an intervention would be considered primarily an intervention at the individual level with minimal crossover into the interpersonal level. One study intervened at the organizational level [32], and no studies were found to intervene at community or political levels. There were a number of studies which were seemingly conducted at a social ecological level beyond the individual but upon closer examination were in fact targeting individuals within broader settings rather than targeting change at a higher social ecological level itself. For example, the “Choose to Move for +(Positive) Living” intervention drew participants from a *community-based* “Stay the Course” obesity support group and sought to determine the influence of psychosocial aspects of the (physical activity and heart healthy living) program on increasing physical fitness, perceived social support and quality of life, and stage of health behaviour change for physical activity. These program's objectives were individual-oriented, and although the intervention appeared to operate as a holistic, community-based program, it did not intervene at the community level [33]. Similarly, the Ke'Ano Ola: Moloka'i's community-based healthy lifestyle program was conducted *in *the community and was based on principles of community-based participatory research. Yet, the intervention targeted individual nutrition education [34]. 

 A number of interventions did not include a theoretical rationale or explanation related to the social aspect of the intervention or program (*N* = 9). Most interventions referenced the stages of change model (or transtheoretical model) and social cognitive theory (social learning theory) (*N* = 4 and *N* = 7, resp.). The typology was developed to identify the way in which the social relational constructs were included in the interventions. The role that researchers considered the social constructs to play in obesity prevention was reflected in the placement of the construct along the intervention pathway. Two of the thirty studies reviewed featured social relational constructs (social networks [30] and social support [35]) as intervention targets. Of the studies which featured social support as the social relational construct, twelve of these operationalized social support as an ancillary resource with the remaining seven studies operationalizing social support as a channel. The studies that featured social cohesion as the social relational construct operationalized it as an ancillary resource. Social networks were mainly operationalized in these studies as a channel to deliver the intervention itself [25, 27, 29]. Two studies included social networks as an ancillary resource [26, 28], and one exceptional study conceptualized social networks as an intervention target [30]. 

## 4. Discussion

 The purpose of this study was to review the types of obesity interventions targeting social relational constructs and characterize the degree to which these interventions have addressed key social relational constructs in intervention design and implementation. Social support was the predominant social relational construct targeted [21, 22, 24, 31–47], treated as a mediator or channel [48], or used as the control treatment in a trial [49]. Social support was not always clearly defined, with a diverse range of social support (peer, family, group, and professional) being delivered either inperson through peer groups or professional therapy or remotely through such tools as handbooks, newsletters, or electronic support messages. The measurement of social support also varied across interventions (e.g., perceived versus actual support). Social support was often assumed to be inherent in any intervention that involved a support group. For example, monthly meetings of overweight/obese individuals who might share their challenges with healthy eating or physical activity were considered to be inherently supportive and equally available to all participants. As a result, many interventions failed to measure whether participants actually received social support. Only four of the 23 studies which focused on social support included a theoretical rationale or evidence for addressing social support in the intervention [22, 30, 33, 42]. The different functions of social support (informational, emotional, tangible, and belonging) were outlined in only two of the 23 studies featuring social support [30, 33]. The nondifferentiation of social support highlights the atheoretical treatment of social support as an agent of change in reducing obesity. Five studies mentioned social support in combination with social cohesion as shared attributes of peer support groups but did not distinguish between these two different social relational constructs by definition or measurement [22, 37, 43, 45, 48]. Overall, social networks were largely limited to methodological applications, as a means of study recruitment or disseminating information related to behavioural change. Little attention was given to the network measures or the effects that social networks might have on health. One exception was Gessell et al.'s [30] study in which they examined the evolution of social networks over the duration of an obesity prevention intervention. In terms of other social relational constructs, there were no studies which discussed social trust, collective efficacy, or social capital. The lack of interventions targeting these higher ecological social network or relational variables suggests that there is still much work to do in translating social capital work into actual interventions, specifically obesity. In addition, there may be a lack of familiarity with, or confidence in the use of, “more complex” social interventions in public health practice. Social support was inconsistently defined, measured, and applied in the current collection of the literature; this might imply that health researchers are differentially receptive to including social support in an intervention, as compared to other social relational constructs. Social support may seem intuitive and most easily intervened on amidst the differing definitions and approaches to measuring social capital; the sophisticated methods of social network analysis; and the vagueness of social cohesion and collective efficacy (and challenges of measurement).

 The social ecological model provides a framework from which to discern and compare the complexity of the different interventions examined in the current review. While intraindividual factors, including beliefs, knowledge, and skills, are important aspects in the behaviour change process, interventions which are limited to targeting change at an individual level fail to address the importance of broader social, physical, economic and political contexts. The breakdown of study types by social ecological level was shown to be pyramid-shaped with the vast majority of studies focused on the individual [22, 24–27, 33, 34, 36–45, 48, 49] and a few interventions that included components which spanned into the interpersonal [23, 28, 30, 31, 35, 46, 47] or organizational realms [21, 29, 50]. Within organizational realms, interventions tended to target making nutritional or physical activity resources available. For example, in a school setting, playgrounds and school yards were made accessible for children to play after end of curricular program, and school canteens were obliged to have fresh fruit and freshly made juices [32]. Another study program modified the cafeteria food service program (the contents of vending machines), and physical education programs [50] and another intervention included implementing short PA breaks during lessons [21]. 

The prominence of individual-level obesity interventions was matched by the greater reliance on theoretical perspectives built on individual psychosocial and behavioral models and constructs. Interventions tended to be driven by theories largely centered on behavioral psychology, including social cognitive theory, the transtheoretical model, and the theory of planned behavior. The lack of social theory in intervention planning limits the development of higher ecological level interventions on obesity. For example, an obesity intervention which is based solely on social cognitive theory would likely lack the breadth to investigate or address the range of environmental factors that might impact person's odds of being obese. 

Furthermore, the frequent reference to self-efficacy in the selected interventions requires additional attention. Self-efficacy—which comprises an individual's motivation, locus of control, and behavioural choices, intentions, and actions with respect to their goals, tasks, and challenges—was often included as a predictor, mediator, or moderator of overweight and obesity risk factors and status. The theoretical emphasis on personal responsibility and control belies the use of concepts related to social, political, and organizational change [5]. This is not to detract from the value of individually oriented theories [9]. However, mounting evidence suggests that innovative strategies for addressing and preventing obesity at a population level should entail theories and approaches that operate from an ecological perspective [51]. 

There were a range of outcomes found in the set of interventions. Obesity-related outcomes included (1) anthropometric indicators, such as body mass index or body fat percentage, (2) physiological measures of cholesterol, blood pressure, and blood sugar, and (3) behavioural risk factors such as physical activity, dietary patterns and knowledge, screen time, sedentary time, and smoking. A number of studies included psychological and psychosocial outcomes, such as depressive symptoms, self-efficacy, and motivation, while some studies also included social indicators, such as social support.

 The conceptualization of a social relational construct as an intervention target would suggest that the researchers view the particular construct as integral to the obesity pathway. Yet, within our sample of interventions, social relational constructs were predominantly incorporated as a channel through which to deliver the intervention, or a nonessential intervention resource. Accordingly, these social relational constructs may be seen as being useful but not amenable characteristic in and of themselves. Although the framing of the rationale of some studies suggests a conceptual emphasis being put on the respective social relational constructs, it is apparent that this emphasis does not carry through in practice. When examining the studies collectively, these findings suggest either (i) a possible stagnation of intervention research that builds on different social relational constructs as they contribute to obesity or (ii) the idea that the conceptualization, implementation, and evaluation of interventions which incorporate social relational constructs and theories beyond the individual are dauntingly complex and inaccessible among researchers. 

 Despite the comprehensiveness of our search strategy, our search criteria may have favored the discovery of smaller scale interventions that would be communicated in more traditional academic outlets. Accordingly, one limitation of our study may have been the potential exclusion of broader policy planning interventions that might target more upstream social political determinants of obesity. Upstream social interventions might consist of one or more social relational constructs or address multiple levels of the social ecological framework. Nevertheless, the lack of interventions on social relational constructs suggests a limited landscape of social relational interventions being implemented or incorporated in broader policy interventions. 

## 5. Conclusion

 To address the problem of obesity, there is a need for public health programs to intervene at social ecological levels beyond the individual. Intervening on interpersonal, organizational, or community levels may be more effective and sustainable in the long term in reducing individual risk of obesity. The apparent lack of social network as opposed to individual support interventions addressing obesity highlights a key gap existing between research and practice. While social epidemiological research has examined the influence of social networks, social capital, and social environments on obesity, this research has yet to be translated into the design of social relational or network interventions that address obesity. While social support may be an important component of such interventions, there is a need to consider more carefully the importance of social relationships and the social environment on the onset and establishment of obesity. The findings of the current study suggest a vast potential for methods and evidence from social health research to further advances in addressing the obesity epidemic.

## Figures and Tables

**Table 1:** Comprehensive overview of intervention studies found pertaining to social relational constructs and obesity.

Article	Social relational constructs			Theory	Social ecological level targeted	Conceptual pathway placement of SRC
	Type	Modality	Measure			
Lubans et al., 2012 [31]	Social support	Text messages	—	Social cognitive theory	Individual/ interpersonal	Resource

Angelopoulos et al., 2009 [32]	Social support	Student workbook and teacher manual including motivational method and strategies (change social influence through modeling, mobilizing social support)	—	Theory of planned behavior	Individual/ interpersonal/ organizational	Resource

Peterson and Ward-Smith, 2012 [33]	Social support	Community-based obesity support group	Social support questionnaire	Transtheoretical model, social comparison theory, and social support	Individual	Channel

Gellert et al., 2010 [34]	Social support	Support group	—	Stage-of-change model	Individual	Resource

Kushner et al., 2006 [35]	Social support	Cohesiveness between owners and pets	Social support and readiness questionnaire	Social learning theory	Individual/ interpersonal	Target

Rimmer et al., 2009 [36]	Social support	Professional advice, brochure information, PA device, telephone consultation, and monthly exercise support group	—	—	Individual	Channel

Stolley et al., 2009 [37]	Social support	Activities to promote group cohesion (ice breaker, potluck dinners, outside activities, and inclusion of friends and family)	Social support for eating and exercise questionnaire	Social cognitive theory, health belief model	Individual	Resource

Hemmingsson et al., 2008 [38]	Social support	PA behavior change booklet, care at obesity unit, and group sessions	—	Transtheoretical model	Individual	Channel

Gold et al., 2007 [48]	Social support	Behaviour therapy with social support lesson; group support	Perceived social support scale	—	Individual	Channel

Anderson et al., 2005 [39]	Social support	*Proposed mediator for changes in HRQOL *	Family social support (Sallis)	Social cognitive theory	Individual	Resource

Gallagher et al., 2006 [40]	Social support	Group sessions (behavioral strategies to elicit social support)	Behavioural processes subscale	Social cognitive theory	Individual	Resource

Pettman et al., 2008 [41]	Social support	Peer group setting incorporating self-management programs, establishing peer support networks; information, shared experiences, and outside interaction	—	Theory of planned behaviour	Individual	Channel

Kiernan et al., 2012 [42]	Social support	Friend and family support for healthy eating and PA	Support subscales and sabotage subscales; general supportive and strained interactions with family and friends subscales; qualitative question on social support	Social support measurement	Individual	Resource

Kalodner and DeLucia, 1999 [43]	Social support	Classmate interaction to facilitate social cohesion and support	—	Behaviour change	Individual	Resource

Casazza and Ciccazzo, 2007 [44]	Social support	Computer-based education; in-person lecture and pamphlets	Social support survey	—	Individual	Resource

Hajek et al., 2010 [45]	Social support	Interactive group sessions (group support components, cohesive and productive environment)	Client feedback questionnaire includes “group support” as potential component participants found most useful	—	Individual	Channel

Yancey et al., 2006 [46]	Social support	Inclusion of close friend or relative	—	Social ecological models	Individual/ interpersonal	Resource

Cousins et al., 1992 [47]	Social support	Emphasized family-oriented approach to health behaviors; manual, inclusion of spouses, and group support	—	—	Individual/ interpersonal	Resource

Williamson et al., 2012 [50]	Social support	Classroom/internet program	Children's dietary social support scale	—	Individual/ organizational	Resource

Leblanc et al., 2012 [49]	Social support	*Structural social support provided by group used in control arm of trial *	—	Health at every size (health-centered approach)	Individual	Channel

Bjelland et al., 2011 [21]	(i) Social support; (ii) social capital	Classmate interaction to facilitate social cohesion and support	(i) Perceived social support from parents, friends, and teachers; (ii) related to people in my area/neighborhood: quality of relationship with peers at school (in + out of classroom)	Social-ecological model	Individual/ organizational	Resource

Lee et al., 2012 [22]	Group cohesion, social support	Intervention group (shared goal, working on team activities, assigning team roles, encouraged to contact each other outside of intervention sessions)	Physical activity group environment questionnaire; social support for eating habits survey	Behavior change; self-efficacy, stage-of-change, and social support	Individual	Channel

De Niet et al., 2011 [23]	Family cohesion	Treatment team (psychologist, dietician, pediatrician, and physiotherapist) led information sessions for parents	Family adaptability and cohesion evaluation scales (FACES) III	Social learning theory	Individual/ interpersonal	Resource

Leahey et al., 2011 [24]	Social cohesion	Participation in group contingent on weight loss	Perceived cohesion scale	—	Individual	Resource

Kim et al., 2004 [25]	Social networks	Participant recruitment through lay health advisors social networks	—	Community-based participatory research	Individual	Channel

Leahey et al., 2012 [26]	Social networks	Media, newsletters, motivational and educational activities, online log, and encouraged team support	Reported social influence for weight loss	Social influence, social learning theory, and social modeling	Individual	Resource

Gorin et al., 2008 [27]	Social networks	Instructional sessions to enhance social support for weight loss efforts	—	—	Individual	Channel

Ashida et al., 2012 [28]	Social networks	Indentified encouragers for dietary behavior	Social influence (enumerated social network members who “played significant role in life during past year” and “have encouraged you to eat more FVs/do PA”)	Social influence	Individual/ interpersonal	Resource

Shaw-Perry et al., 2007 [29]	Social networks	Organized health programming sessions transmitted to children through social structures (home, health class, school cafeteria, and after school)	—	—	Individual/ organizational	Channel

Gessell et al., 2012 [30]	Social networks	Social network evolution over duration of intervention	Social network survey developed to assess changes in social relationships	Social network, social support	Individual/ interpersonal	Target

**Table 2:** Descriptive overview of intervention studies found pertaining to social relational constructs and obesity.

Social relational construct	*N* = 30

Social support	20
Social cohesion	2
Social network	6
Social trust	—
Collective efficacy	—
Social capital	—
*Multiple social relational constructs *	
Social support-social cohesion	1
Social support-social capital	1

Social ecological level targeted	*N* = 30

*Single level target *	
Individual	19
Interpersonal environment	—
Organizational environment	—
Community	—
Political environment	—
*Multiple level target *	
Individual-interpersonal	7
Individual-organizational	3
Interpersonal-organizational	—
Individual-interpersonal-organizational	1

Theory or model	*N* = 30

Health belief model	0 (+1)
Stages of change (transtheoretical model)	3 (+1)
Social learning theory (social cognitive theory)	5 (+2)
Theory of planned behaviour	2
Social support theory	2 (+2)
Social comparison/influence/modeling theory	1 (+3)
Ecological approaches (CBPR, SEM)	3
*Multiple theories, models, or approaches *	5
*No reference to theoretical rationale *	9

Conceptual role of social relational construct	*N* = 30

Intervention channel	16
Ancillary resource	12
Intervention target	2

+ in theory section indicates the addition of partial references of multiple theories. SEM: social ecological model; CBPR: community-based participatory research.
